# Enhancing ‘meaningfulness’ of functional assessments: UK adaptation of the Amsterdam IADL questionnaire

**DOI:** 10.1017/S1041610219001881

**Published:** 2021-01

**Authors:** Gemma Stringer, Iracema Leroi, Sietske A. M. Sikkes, Daniela Montaldi, Laura J. E. Brown

**Affiliations:** 1 Division of Neuroscience and Experimental Psychology, School of Biological Sciences, Faculty of Biology, Medicine and Health, The University of Manchester, Manchester, M13 9PL, UK; 2 School of Medicine, Global Brain Health Institute, Trinity College Dublin, Lloyd Building, Dublin 2, Ireland; 3 Department of Neurology, Alzheimer Center Amsterdam, Amsterdam Neuroscience, Amsterdam University Medical Centers, Amsterdam, The Netherlands; 4 Department of Epidemiology and Biostatistics, Amsterdam University Medical Centers, Amsterdam, The Netherlands; 5 Division of Psychology and Mental Health, School of Health Sciences, Faculty of Biology, Medicine and Health, The University of Manchester, Manchester Academic Health Science Centre, Manchester, M13 9PL, UK

**Keywords:** Instrumental activities of daily living (IADL), mild cognitive impairment (MCI), subjective cognitive decline (SCD), cross-cultural adaptation, Amsterdam IADL Questionnaire (A-IADL-Q)

## Abstract

**Objective:**

Commonly used measures of instrumental activities of daily living (IADL) do not capture activities for a technologically advancing society. This study aimed to adapt the proxy/informant-based Amsterdam IADL Questionnaire (A-IADL-Q) for use in the UK and develop a self-report version.

**Design:**

An iterative mixed method cross-cultural adaptation of the A-IADL-Q and the development of a self-report version involving a three-step design: (1) interviews and focus groups with lay and professional stakeholders to assess face and content validity; (2) a questionnaire to measure item relevance to older adults in the U.K.; (3) a pilot of the adapted questionnaire in people with cognitive impairment.

**Setting:**

Community settings in the UK.

**Participants:**

One hundred and forty-eight participants took part across the three steps: (1) 14 dementia professionals; 8 people with subjective cognitive decline (SCD), mild cognitive impairment (MCI), or dementia due to Alzheimer’s disease; and 6 relatives of people with MCI or dementia; (2) 92 older adults without cognitive impairment; and (3) 28 people with SCD or MCI.

**Measurements:**

The cultural relevance and applicability of the A-IADL-Q scale items were assessed using a 6-point Likert scale. Cognitive and functional performance was measured using a battery of cognitive and functional measures.

**Results:**

Iterative modifications to the scale resulted in a 55-item adapted version appropriate for UK use (A-IADL-Q-UK). Pilot data revealed that the new and revised items performed well. Four new items correlated with the weighted average score (Kendall’s Tau −.388, −.445, −.497, −.569). An exploratory analysis of convergent validity found correlations in the expected direction with cognitive and functional measures.

**Conclusion:**

The A-IADL-Q-UK provides a measurement of functional decline for use in the UK that captures culturally relevant activities. A new self-report version has been developed and is ready for testing. Further evaluation of the A-IADL-Q-UK for construct validity is now needed.

## Introduction

Functional ability refers to an individual’s capacity to complete the everyday tasks necessary for independent living (Lindbergh *et al.*, 2016). It is typically divided into basic activities of daily living, which are simple self-care tasks such as feeding and toileting, and instrumental activities of daily living (IADL), which are more complex, higher order skills such as managing finances and taking medication (Jekel *et al.*, 2015; Lawton and Brody, 1969). The most recent criteria for mild cognitive impairment (MCI) due to Alzheimer’s disease (AD) recognize the presence of subtle problems performing complex functional tasks; however the preservation of independence in functional abilities is a defining criteria (Albert *et al.*, 2011). Nevertheless, difficulties performing IADL in MCI can be predictive of subsequent dementia (Di Carlo *et al.*, 2016; Korolev *et al.*, 2016; Marshall *et al.*, 2015; Sikkes *et al.*, 2011; Tabert *et al.*, 2002). Assessment of subtle change in IADL could therefore provide vital information at the preclinical and prodromal stage of AD to support timely diagnosis and intervention.

Despite the clinical importance of sensitive IADL measurement, current measures of IADL are problematic for a number of reasons. First, although many of these questionnaires are used to assess IADL in people with MCI, they have most often been constructed and validated for people with dementia, and are therefore less sensitive for MCI populations and less able to detect subtle changes in more complex daily activities (Jekel *et al.*, 2015). Second, existing measures fall short in important basic psychometric properties such as reliability and validity (Weintraub *et al.*, 2018). For instance, in a systematic review of 12 IADL scales, only 5 were rated “positive” for how content validity had been assessed (Sikkes *et al.*, 2009). This is important given that the Food and Drug Administration draft guidance published in 2018, emphasizes the need for “meaningful” assessments of functional ability when identifying early AD patients (Food and Drug Administration, 2018). A recent study by Hartry *et al.* (2018) concluded that four commonly used dementia assessment measures do not capture concepts deemed important to patients with mild to moderate AD, suggesting that specific effort is needed to ensure that items are considered conceptually relevant by patients and caregivers. Third, people with cognitive complaints may not have someone to act as an informant, and the majority of questionnaires are informant report, with only a small number of self-report options available. Even fewer questionnaires offer both options (Jekel *et al.*, 2015).

Another issue with many IADL questionnaires currently in use is that they are outdated and do not include modern activities such as using a computer (Sikkes *et al.*, 2012). This is particularly relevant given the growing use and importance of technology and computers in the lives of older adults. For example, adults aged 65 years and over have shown the largest increase in online shopping compared to all other age groups over the past decade, rising from 16% within the age group in 2008 to 48% within the age group in 2018 (Office for National Statistics, 2018). The assessment of everyday technology use has been shown to provide sensitive measures of early change in functional ability (Hedman *et al.*, 2018; Malinowsky *et al.*, 2010; Rosenberg *et al.*, 2009). So, including modern activities such as the use of computers and mobile phones in IADL assessments could potentially improve sensitivity to subtle change at an early stage in cognitive decline (Jekel *et al.*, 2015).

The Amsterdam IADL Questionnaire (A-IADL-Q) was developed with the aim of providing a more up-to-date overview of the IADL used in a technologically advancing society (Sikkes *et al.*, 2012). The A-IADL-Q was originally developed in the Netherlands and has since been translated and culturally adapted for use in 12 languages (https://www.alzheimercentrum.nl/professionals/amsterdam-iadl-translations/) (Dubbelman *et al.*, 2019; Facal *et al.*, 2018). However, to date, it has not been culturally adapted and validated for use in the U.K. Therefore, the content of the questionnaire may not reflect the cultural norms and everyday behaviors of the U.K. population. In addition, the A-IADL-Q does not currently have a self-report version, which limits its use to people with an informant, or comfortable using an informant.

The aim of this study was therefore to complete a cross-cultural adaptation of the informant-basedA-IADL-Q for use in the UK (the A-IADL-UK) and to develop a self-report version. The objectives were to: (1) assess the face and content validity of the translated questionnaire with lay and professional stakeholders and generate candidate new items to represent culturally important IADL; (2) further adapt the questionnaire based on the relevance of all candidate items to older adults in the UK; and (3) pilot the A-IADL-UK in a group of older adults with subjective cognitive decline (SCD) and MCI, to assess the relevance and sensitivity of new items and explore associations with measures of cognition and function. We hypothesize that higher levels of impairment measured by the A-IADL-UK will be associated with higher levels of cognitive and functional impairment on these existing measures.

## Method

### Design

**Figure 1.**
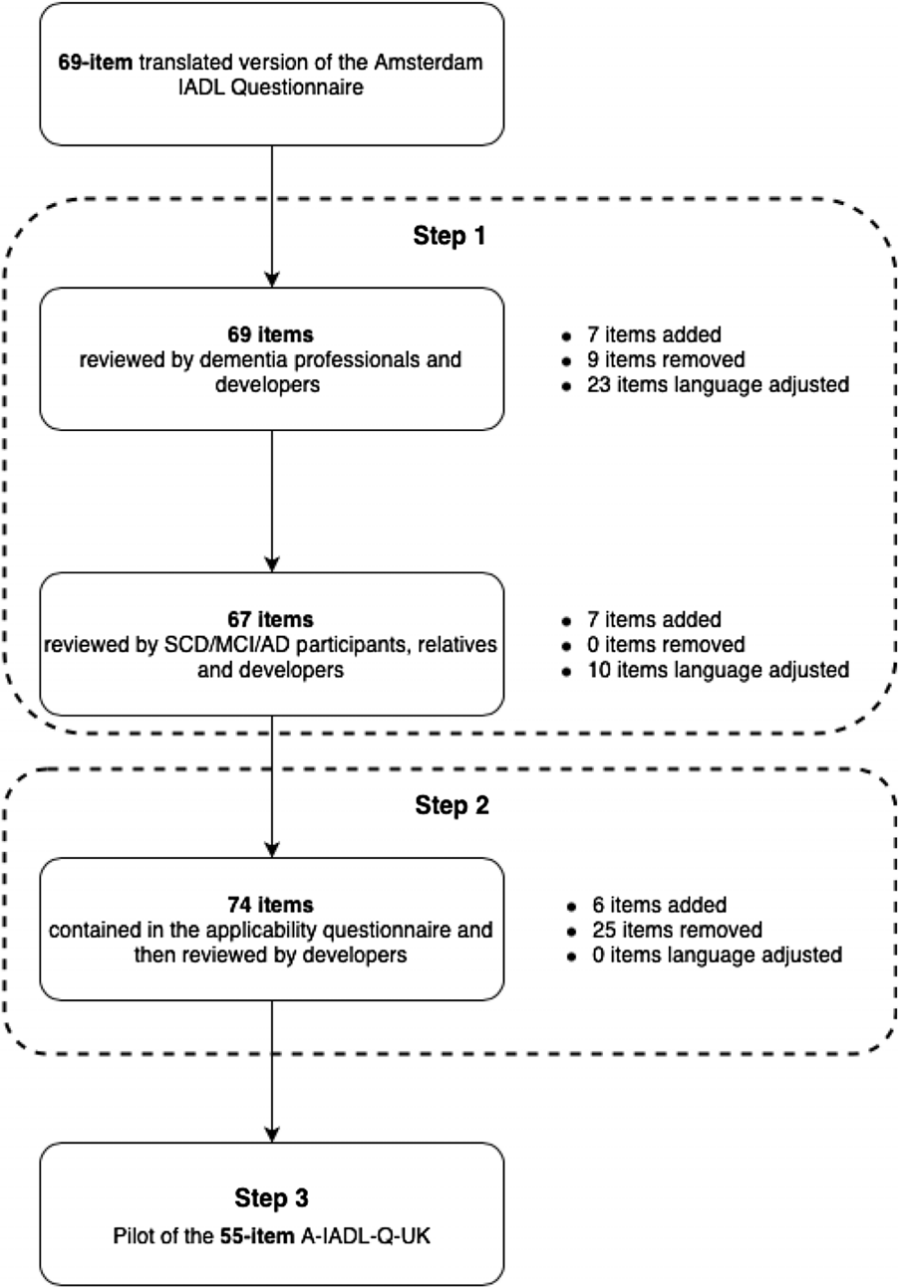
Flowchart detailing the outputs from the three steps of the A-IADL-Q-UK item adaptation process. See Appendix 1 for full details of the changes made to each item at each step.

This was an iterative mixed method cross-cultural adaptation of the A-IADL-Q undertaken in community settings in England involving a three-step design (Figure 1). In step 1, interviews and focus groups were conducted with lay and professional stakeholders to assess the face and content validity of the items. In step 2, a questionnaire was administered to older adults to measure the frequency of daily activities. In step 3, the questionnaire adapted from the results of steps 1 and 2 was piloted.

### Participants

Five groups of participants took part in the study, across three steps (Table 1).

**Table 1.** Overview of all participant demographics across three steps

	step 1			step 2	step 3
	dementia professionals (*n* = 14)	scd/mci/ad participants(*n* = 8)	relatives (*n* = 6)	older adults^a^ (*n* = 92)	scd/mci participants (*n* = 28)
Age (years)	N/A	78.30 (5.95)	68.87 (13.13)	73.87 (5.53)	72.46 (4.04)
Gender (% women)	11 (79)	4 (50)	4 (67)	62 (71)	18 (64)
Ethnicity, white British	N/A	N/A	N/A	87 (95%)	N/A
Years of formal education	N/A	N/A	N/A	Left school before 16 (29%)	13.21 (3.24)
ACE III total score	N/A	N/A	N/A	N/A	93.54 (5.49)
ECog total score	N/A	N/A	N/A	N/A	1.60 (.68)
TMT B	N/A	N/A	N/A	N/A	77.96 (41.31)
DSB	N/A	N/A	N/A	N/A	7.79 (2.41)

Data are presented as mean (SD), or *n* (%).

Abbreviations: ACE III, Addenbrooke’s Cognitive Examination-III (ACE III); ECog, measurement of everyday cognitive function; TMT B, trails making test B; DSB, digit span backwards task.

a Demographic data were not completed by five of these participants.

Dementia professionals were recruited by email and personal contacts. In order to be included, they had to work with older adults and have experience of diagnosing dementia and/or conducting cognitive or functional assessments with people with dementia. The final group of dementia professionals (*n* = 14) consisted of five consultant old age psychiatrists, four trainee consultant psychiatrists, three dementia research nurses, and two later life occupational therapists from the northwest of England.

Older people (over the age of 65 years) with SCD, MCI, and mild dementia due to AD were recruited for steps 1 and 3 through memory clinics; the UK dementia research registry “Join Dementia Research” (a national web-based service for participation in dementia studies); step 3 participants were also part of another study called Software Architecture for Mental Health Self-Management (SAMS) (Stringer *et al.*, 2018). Participants with dementia and MCI diagnoses were referred with a diagnosis already made by qualified memory specialists. Participants with self-reported worries about their memory were identified as SCD if they indicated on a scale of functional capacity – the Everyday Cognition Scale (ECog) (Farias *et al.*, 2008) that they were “concerned they have a memory or other thinking problem” and their total score on this scale was >1.436. This cutoff score corresponds to the upper 95% confidence interval of the mean total ECog scores from a sample of healthy control participants who indicated that they were *not* “concerned they have a memory or other thinking problem” (Stringer *et al.*, 2018). Participants who did not meet this criterion for SCD were not eligible to take part. The SCD/MCI/AD participants and relatives in step 1 were recruited using the “sampling to redundancy” criterion, that is, interviewing participants until no new themes emerge (Streiner, 2008).

Older adults (over the age of 65 years) who were cognitively healthy were recruited for step 2 through Join Dementia Research and local community groups in the Greater Manchester area.

All participants were included if they had the capacity to consent and were able to communicate verbally in English. Individuals with any severe physical or mental difficulties were not eligible for the study.

### Description of the parent instrument

The A-IADL-Q consists of 70 items, plus 6 additional sociodemographic questions that can be added or adapted per study, and is completed by an informant of the patient. IADL are divided into seven categories: household duties, domestic appliances, household budget, work, computer, devices, and leisure time/other. Participants are asked if they have completed the activity in the previous 4 weeks and, if yes, their difficulty performing the activity is rated on a 5-point Likert scale ranging from “no” difficulty in performing the task to “no longer able to perform this task”. The A-IADL-Q has been shown to have good content validity and test–retest reliability (Sikkes *et al.*, 2012) and good construct validity (Sikkes *et al.*, 2013a). A recently developed 30-item A-IADL-Q short version (A-IADL-Q-SV) maintained the psychometric quality of the original A-IADL-Q (Jutten *et al.*, 2017).

Items are adapted based on the respondent’s answers. For example, further questions about computer use are only asked if the patient answers that they use a computer. The total score on the questionnaire is calculated using an item response theory (IRT) method of scoring, with lower scores indicating poorer performance (Sikkes *et al.*, 2013b). IRT assumes that ordered categorical item responses represent an underlying construct or “latent trait” (Embretson and Reise, 2000). This construct for the A-IADL-Q is IADL functioning ranging from ability to disability.

### Procedures

The study was approved by the Health Research Authority – National Research Ethics Service England in accordance with the Declaration of Helsinki, and all participants signed informed consent to participate. A 69-item paper-based draft version of the UK informant questionnaire was created using a combination of items from existing US and Australian culturally validated versions of the A-IADL-Q. The self-report version was created by rephrasing each item from third person to second person (e.g. “…did they use a computer?” was rephrased “…did you use a computer?”). The 69-item draft version was then culturally adapted through the following three-step process (Figure 1).

### Step 1. Dementia professionals’, SCD/MCI/AD and relatives’ review

The first step involved reviews by two groups of people: (1) the dementia professionals and (2) people with SCD, MCI, or mild dementia due to AD and relatives of people with MCI and dementia. Participants were presented with the 69 IADL items in the form of a Likert scale questionnaire that asked participants to rate how often they did each activity. The questionnaire included five example questions to illustrate the wording of the final version (e.g. “In the past four weeks did you use a sat-nav?”). During face-to-face interviews or small focus groups, dementia professionals commented on the clarity and appropriateness of the activity wording and the relevance of the activities to older adults in the UK, and they also suggested relevant new activities. Written notes and audio recordings were taken throughout. A summary of the findings was discussed in a developer review with the developer of the original scale (Sietske Sikkes) via video conference, where suggested changes were considered and decisions were made for each item in preparation for the next stage. This consultation allowed for constructive feedback to ensure that the integrity of the scale was maintained. The changes made following the developer review resulted in a 67-item version.

SCD/MCI/AD participants and relatives completed the 67-item version individually and were asked to think aloud throughout. SCD/MCI/AD participants completed the self-report version, and relatives completed the informant report version. All participants were also asked to suggest any new activities that were not covered in the questionnaire but were relevant to their daily lives. Comments were audio-recorded and written notes were made. To improve the clarity of items, we made minor iterative changes to the layout and wording of questions in response to feedback from individual participants. More substantial changes to the actual activities, and any deletions or additions, were made following a second developer review.

### Step 2. Applicability questionnaire

The two-stage review process in step 1 resulted in a 74-item applicability questionnaire comprising 60 original items, 33 modified original items, and 14 new items. In step 2, to assess relevance, the frequency of the activities in the 74-item version was measured using a postal questionnaire. Participants were asked to rate how frequently they completed the activities on a 6-point Likert scale from “most days” to “never”. Participants were also able to suggest new activities and provide additional comments. Two follow-up questions (“Did they buy the correct amounts?” and “Did they buy the correct items?”) were excluded from the applicability questionnaire because they were not compatible with a frequency rating. Decisions about changes to the excluded items were based on the developer discussion. Paper copies of the 72-item questionnaire were distributed to 140 older adults over the age of 65 years who did not have a diagnosis of dementia. Completed questionnaires were returned by post.

Analysis of the responses to the step 2 applicability questionnaire considered the mean and median responses for each activity. Activities with a median score of >4 (corresponding to activities not undertaken in the past year or ever) were considered candidates for removal. New activities with a median score <2 (corresponding to activities done every day or one to three times per week) were considered candidates for inclusion. Items with >6 missing answers, or that were difficult for participants to complete based on observations in the completed versions or notes made by the participants, were also considered candidates for exclusion. In addition, information about item performance from the development of the A-IADL-Q-SV was used to help guide decisions about the inclusion or exclusion of some items. A discussion of the median analysis and decisions about changes were completed in a third developer review, which led to a final 55-item version being created.

### Step 3. Pilot of the A-IADL-Q-UK

In step 3, a 55-item electronic version of the questionnaire was piloted to assess the relevance and perceived difficulty of the new items and to measure overall functional impairment. The UK version (A-IADL-Q-UK) comprising the items developed in steps 1 and 2 was administered electronically using Qualtrics software Version New QTrial 2015 to 31 older adults with either SCD or a diagnosis of MCI. Participants were sent a link to the questionnaire via email (Copyright © [2017] Qualtrics. Qualtrics and all other Qualtrics product or service names are registered trademarks or trademarks of Qualtrics, Provo, UT, USA. https://www.qualtrics.com). A reminder telephone call was made if the questionnaire had not been completed after 5 days. The 55-item version included 9 “new items”, 4 items that were completely new, and 5 items for which the language or meaning had been adjusted significantly and meant that existing item characteristics could no longer be used, for example, “using a coffee maker” became “making a cup of tea or coffee”. As no item characteristics were yet available for the new items and due to sample size, it was not possible to use IRT analysis on the step 3 pilot data; therefore, scores for this were calculated using the weighted average (WA). This alternative scoring approach was previously tested for the Amsterdam IADL and is currently used in clinical practice due to a high concordance with the IRT scoring. WA was calculated by dividing total IADL score by the number of items endorsed. The following scoring method was then applied 100 − (WA*25). Higher scores indicate greater functional impairment.

### Step 3: Instruments

Descriptive measures of global cognitive status were obtained using the Addenbrooke’s Cognitive Evaluation (ACE) III (Hsieh *et al.*, 2013): a concise neuropsychological assessment of cognitive functions commonly used in the U.K. with validated cutoff scores for MCI and dementia. The battery includes five cognitive subdomains: attention, memory, verbal fluency, language, and visuospatial abilities, which provide a cognitive score out of a maximum of 100 (a higher score indicates more intact cognition). The ACE III was selected to investigate the relationship between the A-IADL-UK and global cognitive status.

In order to focus on critical areas of executive functioning, we selected the digit span backwards (DSB) test and trails making test (TMT) B (Lezak *et al.*, 2012). TMT B is a measure of executive abilities including set-shifting and mental flexibility, and a longer time on this test represents a higher level of impairment. DSB is an executive task particularly dependent on working memory where a higher score represents less impairment. TMT B and DSB are known to be sensitive to age (Lara *et al.*, 2013). Participants’ scores on the DSB and TMT B were compared with a larger set of tests administered. Participants’ scores on TMT B and DSB appeared to have no ceiling effects, and examination of longitudinal data from the SAMS study indicated that participants were not improving on these tests over time.

Subjective ratings of cognitive and functional capacity were obtained using the self-report and informant version of the ECog (Farias *et al.*, 2008). This assessment requires the individual or their informant to rate current functional ability compared to ability 10 years previously. The 39-item questionnaire assesses cognitively based functional items, across 6 neurological domains: memory, language, visuospatial abilities, planning, organization, and divided attention. Scores range from 1 (“Better or no change”) to 4 (“Consistently much worse”). The A-IADL-UK was compared with the ECog to test whether the two scales were measuring the same construct.

Participants who self-reported (*n* = 7) did so on the A-IADL-UK and the ECog. Participants self-reported if they did not have someone who could act as an informant. For all other participants (*n* = 21), their informant provided responses to these scales. Informants were defined as people who lived with, cared for (if required), or had at least weekly contact with the participant.

### Step 3: Statistical analysis

Statistical analyses were performed using SPSS version 22. Since most datasets were not normally distributed (Kolmogorov–Smirnov goodness of fit test *p* < 0.05), nonparametric tests were used. Correlations were investigated using Kendall’s tau-b correlation coefficient because the approximations are better for small sample sizes (Arndt *et al.*, 1999). The significance level was set at *p* < 0.05, unless indicated otherwise. Due to an administrative error in the questionnaire administered to participants in step 3, answers to the question “Did they use technology?” were not included in the WA scores. To investigate construct validity, correlations were considered between the A-IADL-UK and a number of other neurological tests selected from a larger set of tests that were administered.

## Results

Details of changes to all items in the questionnaire for each step of the cultural adaptation, including reasons for removing and adding items, adjustments to language, decisions from the developer reviews, and the final item wording, can be found in Supplementary Appendix 1. For a summary of the numbers of questions added, removed, and amended, see Figure 1.

### Step 1. Dementia professionals’, SCD/MCI/AD and relatives’ review

Seven items were added and nine items were removed based on suggestions from the dementia professionals and discussion with the developer. Of the seven items added, two were completely new items suggested by the dementia professionals and the other five items were added by the developer based on what was contained in the original Dutch version. Items were removed based on the suggestions from the dementia professionals for a variety of reasons: two were thought to be confusing; four were considered analogous to other activities, for example, “booking a trip on the internet” was removed as it was considered similar to “booking holidays” and “buying on the internet” was already covered in another question; and three were judged as outdated, for example, “using cheques”. Suggestions of changes in language were undertaken for 26 items, for example, the term “operating” was changed to “using” for 10 items as this was thought to be a more common term. Some items that were considered to be confusing or incongruous by dementia professionals were retained to see how the other participants responded to these items.

Following suggestions from the SCD/MCI/AD participants and the relatives, a total of 7 new items were added and 10 items were adjusted for language. The main reasons for adjusting the language of items were to add more common terms, for example, “electronic banking” was replaced with “internet banking” and to clarify the meaning of questions, for example, not all participants knew what a smartphone was, so this was changed to “using a mobile phone to go on the internet”. Items that dementia professionals highlighted as confusing or incongruous were the same ones designated this way by the SCD/MCI/AD participants and relatives. Again, these items were retained at this stage to seek further data on how people engage with them.

### Step 2. Applicability questionnaire

Of the 140 questionnaires distributed, 92 (65.7%) were returned. Nineteen items were identified as candidates for exclusion and 53 were candidates for inclusion on the basis of the median frequency that each IADL were completed. Following a third developer review, 6 items were added, 24 items were removed, and 2 items were adjusted for language, resulting in a final item count of 55 for use in the pilot.

### Step 3. A-IADL-Q-UK pilot

For the pilot, 31 questionnaires were distributed via email link. An email reminder was sent after 2 weeks. A total of 28 questionnaires (90.3%) were completed. Twenty-one of these participants (75%) had somebody who could act as an informant and seven participants (25%) provided the information themselves in the form of self-report. Although participants with SCD obtained higher mean A-IADL-Q-UK scores (*n* = 17, mean = 98.64, SD = 2.05) than MCI participants (*n* = 11, mean = 90.28, SD = 15.37), this difference was not significant (*u* = 76.50, *p* = 0.430) (Weighted total including new items.).

### Response characteristics of new items

The number of participants endorsing each response to the nine new items and the correlation analyses for the new items in relation to total score are shown in Table 2. The new and revised items performed well: at least half of the participants had completed each activity in the previous 4 weeks and all activities were perceived as either slightly more, more, or much more, difficult by at least one participant per item. When broken down by subgroup (informant report version and self-report version), because of the small numbers (particularly in the self-report subgroup), for some items there was no variation in the responses given to specific items within one of the subgroups (e.g. with none of the participants in one subgroup finding the activity more difficult: see supplementary Table 1). Therefore, the analysis was focused on both groups as a whole. “Making a cup of tea or coffee” and “using keys” were the most frequently performed activities. “Reading” was another activity completed by the majority of participants and over 10% (3) of participants found this more difficult to some degree. Despite low numbers of participants completing the activities, “recording a television program”, “completing household paperwork”, and “maintaining the garden” were each seen as being slightly, more, or much more difficult by three or more participants. Most participants did not find “making a cup of tea or coffee”, “using the hob”, “using the grill”, “using keys”, or “looking after family” more difficult.

**Table 2.** Number (and %) of participants endorsing each response to new items, and correlation coefficients of new items with weighted average score

	relevance		difficulty			kendall’s tau-b correlation coefficients of new items and weighted average score (without new items)	
item	did not do activity in previous 4 weeks or have never done activity	completed activity in previous 4 weeks	did not find the activity more difficult	found activity slightly more difficult	found activity more/much more difficult	correlation with weighted average score without new items	*p* value
Making a cup of tea or coffee^a^	0 (0.0)	28 (100.0)	27 (96.4)	1 (3.6)	0 (0.0)	−0.25	0.12
Using the hob^a^	2 (7.1)	26 (92.9)	24 (92.3)	2 (7.7)	0 (0.0)	−0.08	0.63
Using the grill^b^	11 (39.3)	17 (60.7)	16 (94.1)	1 (5.9)	0 (0.0)	−0.35	0.10
Completing household paperwork^a^	6 (21.4)	22 (78.6)	19 (86.4)	2 (9.1)	1 (4.5)	−0.50	0.01
Recording a TV program^a^	10 (35.7)	18 (64.3)	15 (83.3)	2 (11.1)	1 (5.6)	−0.57	0.01
Using keys^a^	0 (0.0)	28 (100.0)	27 (96.4)	0 (0.0)	1 (3.6)	−0.27	0.09
Reading^b^	1 (3.6)	27 (96.4)	24 (88.9)	2 (7.4)	1 (3.7)	−0.45	0.01
Maintaining the garden^b^	6 (21.4)	22 (78.6)	18 (81.8)	2 (9.1)	2 (9.1)	−0.39	0.03
Looking after family^b^	13 (46.4)	15 (53.6)	14 (93.3)	1 (6.7)	0 (0.0)	−0.37	0.10

a Classified as new because of significant changes to language and/or meaning.

b Completely new item.

The items “using the grill” and “looking after family” were performed least frequently by participants. Reasons for not using the grill were mixed but most participants reported that they had never used it or had no need to. The majority of participants reported that they did not “look after family” because they lived too far away, did not have any family to look after, or had never done it.

There was a significant correlation between the WA score and four of the new items: “completing household paperwork”, “recording a television program”, “reading”, and “maintaining the garden”. The remaining five new items were not significantly correlated with the total score.

### Exploratory validation analysis of the A-IADL-Q-UK and other neuropsychological tests

Table 3 shows the correlations between the WA score of the A-IADL-Q-UK (including the new items) with age and clinical measures. The A-IADL-Q-UK total score did not correlate with age. All correlations with cognitive and functional measures were in the expected directions: these were significant for DSB and ECog and nonsignificant for ACE III and TMT B. When broken down by subgroup (informant version and self-report version), not all of these patterns held (see supplementary Table 2).

**Table 3.** Means and Kendall’s tau-b correlation coefficients of weighted average scores (including the new items) of the A-IADL-Q-UK with clinical measures and demographics

measure	*N*	mean (sd)	weighted average score with new items (kendall’s tau-b)	*p* value
Demographic data				
Age	28^†^	72.64 (4.40)	−.13 (−.45, .19)	.36
Cognitive functioning				
ACE III^‡^	28	93.54 (5.49)	.11 (−.28, .44)	.46
DSB^§^	28	7.79 (2.41)	.38 (.50, .62)	.01
TMT B^‖^	28	77.96 (41.31)	−.04 (−.37, .28)	.77
Everyday functioning				
ECog^¶^	28	1.60 (.68)	−.46 (−.67, −.22)	.00

† Demographic data were not completed by 5 of these participants.

‡ ACE III = Addenbrooke’s Cognitive Examination-III (ACE III).

§ DSB = Digit Span Backwards Task.

‖ TMT B = Trails Making Test B.

¶ ECog = Measurement of Everyday Cognitive Function.

## Discussion

This development of the A-IADL-Q-UK enhanced the conceptual and cultural relevance of the original version of the questionnaire for an older adult UK population. We assessed face and content validity by utilizing the views of people with cognitive impairment, their relatives, UK older adults, and dementia professionals. This essential step improves the clinical meaningfulness of functional assessments for people with SCD and MCI in the UK. The informant version was adapted and a self-report version of the questionnaire was developed. Modifications to the scale included adding 20 items, removing 34, and adjusting the language of 33 items, resulting in a 55-item adapted version. This version of the scale is ready for a full-scale validation.

This cross-cultural validation is important because mere translation of an instrument does not always account for cultural and ethnoracial disparities (Beaton *et al.*, 2000). Frequently used IADL instruments often include culturally specific activities such as balancing a chequebook (Dubbelman *et al.*, 2019). We found that activities such as using a coffee maker and a dishwasher were not common practice among current older adults in the UK, and therefore amending or removing these items enhances the validity of the measure for the UK audience.

Four of the nine new items correlated with the total score of the A-IADL-UK. Interestingly, these items were also the ones that most participants felt were more or much more difficult, suggesting that these items are more sensitive to functional impairment in this sample of participants with SCD and MCI. This is in line with research by Marshall *et al.* (2015) who found that the item “assembling tax records” discriminated between healthy and MCI participants, and that lower scores on a “paying bills/balancing checkbook” item predicted progression from healthy to MCI. However, they also found that “heating water and turning off the stove” was sensitive to functional change, whereas in our sample most participants did not find a similar item “using the hob” more difficult. This discrepancy could be due to the smaller sample size in the current study or because the question in the Marshall *et al.* study asks specifically about remembering to turn off the stove.

Construct validity of the new A-IADL-UK was explored by considering correlations with age and other clinical measures. The exploratory analysis of the WA score including the new items found no correlation with age. This is in line with work by Sikkes *et al.* (2013a), who found small but significant correlations with age in the original questionnaire. This is important as it suggests that the questionnaire can be used without normative data for age. Scores on the A-IADL-Q-UK were significantly correlated with another measure of everyday functioning: the ECog. This demonstrates good convergent validity. Associations with ACE, DSB, and TMT B were all in the expected direction. However, the association with ACE and TMT B was nonsignificant and very weak. Previous literature in this area is mixed. Some studies have found that informant and self-reported measures typically yield small or no associations with executive processes (Aretouli and Brandt, 2010; Jefferson *et al.*, 2006; Plehn *et al.*, 2004). In a more recent study, a longer time spent on TMT B was associated with a lower score on the A-IADL-Q, and a model incorporating DSB indicated a satisfactory fit when testing the relationship between change in IADL and change in memory functioning (Koster *et al.*, 2015). There are limited studies exploring the relationship between the ACE III and IADL measures; research that has been done suggests that the ACE III is sensitive to everyday functioning (Giebel and Challis, 2017; Hsieh *et al.*, 2013; Scally, 2016), but this is based on small sample sizes. In a more recent study, the ACE III was related to functional impairment across a number of dementia syndromes (So *et al.*, 2018). However, none of these studies have looked for a relationship between the ACE III and functional ability in people with subjective or mild cognitive decline. Future studies with larger samples, including those with subjective and mild cognitive decline, will enable further investigation of construct validity. What is most important, however, is the predictive validity of the A-IADL-UK and how well it can predict a person’s ability to function in the real world. Moore and colleagues argue that predictive validity is more important than comparison to normative data, because it shows whether an instrument can predict competency in actual daily life (Moore *et al.*, 2007). Future validation of the A-IADL-Q-UK should compare scores with observed behavior of daily activities inside the individual’s home.

Although there was no significant difference between the scores of participants with SCD and MCI, it is notable that scores for the MCI group were numerically higher than the SCD group. This is in line with findings from the Spanish adaptation of the A-IADL-Q by Facal *et al.* (2018) and provides additional tentative evidence for the validity of the scale as a measure of functional impairment. Future research is now needed to compare scores between these groups in studies with larger sample sizes.

Limitations of the current study include the small sample size and low statistical power for the step 3 pilot. The sample size for step 3 was based on pragmatics such as time and budget constraints and was not intended to be fully powered. In addition, because this sample was part of an ongoing study, the inclusion criteria were set by that study, and therefore cognitively healthy participants and individuals with dementia were not included, even though they were included in step 1 and step 2. This pilot study was a first step in assessing the reliability and validity of the A-IADL-UK, and the results presented are exploratory and further testing is recommended as a next step. The majority of participants in steps 1 and 3 were recruited from the Greater Manchester area of the UK, this means that the study has a low geographical reach. The study also has a limited cultural reach as the majority of participants (95%) in the step 2 applicability questionnaire were white British, which is not reflective of the full ethnic breakdown of the UK (Office for National Statistics, 2011). A further limitation is that ethnicity was not recorded for participants in steps 1 and 3. However, the dementia professionals in step 1 would have knowledge of a wider and more diverse client base which may mitigate some of these issues.

The assessment of face and content validity is an important psychometric property often lacking in the development of existing IADL scales (Sikkes *et al.*, 2009). A major strength of the current study was that the process for assessing face and content validity was detailed and thorough, and this is reflected in the use of a sampling to redundancy criterion in step 1 meaning all suggestions from participants were considered until no new information emerged. A further strength is the inclusion of a range of stakeholders, including people with cognitive impairment and relatives of people with MCI and dementia. To date relatively few assessments of content validity, utilize the knowledge and expertise of patients and caregivers, which often solely relies on the judgments of clinicians (Connell *et al.*, 2018). In addition, we compared the instrument to another IADL measure (ECog) administered to the same participant group, enabling a direct comparison between the two IADL instruments.

In summary, in this first UK adaptation of the A-IADL-Q, we developed the informant version and produced the first self-report version, demonstrating the face validity and content validity of the measure. A comprehensive review of the measure was undertaken and included people with cognitive impairment and their relatives, as well as dementia professionals and cognitively healthy older adults. A self-report version of the questionnaire will allow people without an informant to provide information about their ability to complete the IADL. The next step is to determine item characteristics for the new items, so that final decisions can be made about which ones to include in the A-IADL-Q-UK. This will require further data analysis from a larger sample. Further quantitative testing of the A-IADL-UK on a larger sample will enable assessment of reliability and validity and a full examination of internal consistency and measurement bias.

## Supplementary material

For supplementary material accompanying this paper visit https://doi.org/10.1017/S1041610219001881. click here to view supplementary material
