# Supplementary material for: Enhancing ‘meaningfulness’ of functional assessments: UK adaptation of the Amsterdam IADL questionnaire
Source: Int Psychogeriatr. 2020 Apr 15;33(1):39–50. doi: 10.1017/S1041610219001881 (PMC8482374; doi:10.1017/S1041610219001881)
Supplement: Supplementary file 1 [file S1041610219001881sup.zip › S1041610219001881sup002.docx]

| Supplementary table 1. Number (and %) of participants endorsing each response to new items, and correlation coefficients of new items with weighted average score split by self-report and informant report. | | | | | | | | |
| --- | --- | --- | --- | --- | --- | --- | --- | --- |
|  | Relevance | | | Difficulty | | | Kendall’s tau-b correlation coefficients of new items and weighted average score (without new items) | |
| Item | Did not do activity in previous 4 weeks or never done activity  (informant report / self-report) | Completed activity in previous 4 weeks (informant report / self-report) | Did not find the activity more difficult (informant report / self-report) | | Found activity slightly more difficult (informant report / self-report) | Found activity more/much more difficult  (informant report / self-report) | Correlation with total score without new items (informant report / self-report) | p value (informant report / self-report) |
| Making a cup of tea or coffee* | 0 (0.0) / 0 (0.0) | 21 (100.0) / 7 (100.0) | 20 (95.2) / 7 (100.0) | | 1 (4.8) / 0 (0.0) | 0 (0.0) / 0 (0.0) | -.32 / 1.0 | .10 / N/A |
| Using the hob* | 1 (4.8) / 1 (14.3) | 20 (95.2) / 6 (85.7) | 18 (85.7) / 6 (85.7) | | 2 (9.5) / 0 (0.0) | 0 (0.0) / 0 (0.0) | -.12 / N/A | .53 / N/A |
| Using the grill^‡^ | 7 (33.3) / 4 (57.1) | 14 (66.7) / 3 (42.9) | 13 (61.9 ) / 3 (42.9) | | 1 (4.8) / 0 (0.0) | 0 (0.0) / 0 (0.0) | -.38 / N/A | .11 / N/A |
| Completing household paperwork* | 6 (28.6) / 0 (0.0) | 15 (71.4) / 7 (100.0) | 13 (61.9) / 6 (85.7) | | 2 (9.5) / 0 (0.0) | 0 (0.0) / 1 (14.3) | -.50 / -.55 | .03 / .13 |
| Recording a TV program* | 6 (28.6) / 4 (57.1) | 15 (71.4) / 3 (42.9 ) | 12 (57.1 ) / 3 (42.9 ) | | 2 (9.5) / 0 (0.0) | 1 (4.8) / 0 (0.0) | -.61 / N/A | .01 / N/A |
| Using keys* | 0 (0.0) / 0 (0.0) | 21 (100.0) / 7 (100.0) | 21 (100.0) / 6 (85.7) | | 0 (0.0) / 0 (0.0) | 0 (0.0) / 1 (14.3) | N/A / -.55 | N/A / .13 |
| Reading^‡^ | 1 (4.8) / 0 (0.0) | 20 (95.2) / 7 (100.0) | 19 (90.5) / 5 (71.4) | | 0 (0.0) / 2 (28.6) | 1 (4.8) / 0 (0.0) | -.32 / -.71 | .01 / .05 |
| Maintaining the garden^‡^ | 4 (19.0) / 2 (28.6) | 17 (81.0) / 5 (71.4) | 15 (71.4) / 3 (42.9) | | 1 (4.8) / 1 (14.3) | 1 (4.8) / 1 (14.3) | -.14 / -.84 | .50 / .05 |
| Looking after family^‡^ | 13 (61.9) / 0 (0.0) | 14 (66.7) / 1 (14.3) | 13 (61.9) / 1 (14.3) | | 1 (4.8) / 0 (0.0) | 0 (0.0) / 0 (0.0) | -.38 / N/A | .10 / N/A |
| *classified as new because of significant changes to language and/or meaning  ^‡^completely new item  N/A – all participants in the group gave the same response so there is no variation in the data | | | | | | | | |
