# Supplementary material for: Enhancing ‘meaningfulness’ of functional assessments: UK adaptation of the Amsterdam IADL questionnaire
Source: Int Psychogeriatr. 2020 Apr 15;33(1):39–50. doi: 10.1017/S1041610219001881 (PMC8482374; doi:10.1017/S1041610219001881)
Supplement: Supplementary file 1 [file S1041610219001881sup.zip › S1041610219001881sup003.docx]

| Supplementary table 2. Means and Kendall’s tau-b correlation coefficients of weighted average scores (including the new items) of the A-IADL-Q-UK with clinical measures and demographics split by self-report and informant report. | | | | |
| --- | --- | --- | --- | --- |
| Measure | N  (informant report /  self-report) | Mean (SD)  (informant report / self-report) | Weighted average score with new items (Kendall’s tau-b)  (informant report /  self-report) | p value  (informant report /  self-report) |
| Demographic data |  |  |  |  |
| Age | 21 / 7 | 72.1 (3.66) / 74.2 (6.20) | -.129 / .000 | .451 / 1.00 |
| Cognitive functioning |  |  |  |  |
| ACE III^‡^ | 21 / 7 | 94.71 (4.60) / 90.00 (6.78) | -.023 / .264 | .895 / .428 |
| DSB^§^ | 21 / 7 | 8.14 (2.39) / 6.71 (2.29) | .281 / .474 | .111 / .154 |
| TMT B^‖^ | 21 / 7 | 80.24 (42.55) / 71.14 (39.63) | -.011 / .103 | .948 / .754 |
| Everyday functioning |  |  |  |  |
| ECog^¶^ | 21 / 7 | 1.66 (.76) / 1.41 (.28) | -.578 / .000 | .001 / 1.00 |

‡ACE III = Addenbrooke’s Cognitive Examination-III (ACE III); §DSB = Digit Span Backwards Task; ‖TMT B = Trails Making Test B; ¶ECog = Measurement of Everyday Cognitive Function.
